# Supplementary material for: LARP7-like protein Pof8 regulates telomerase assembly and poly(A)+TERRA expression in fission yeast
Source: Nat Commun. 2018 Feb 8;9:586. doi: 10.1038/s41467-018-02874-0 (PMC5805695; doi:10.1038/s41467-018-02874-0)
Supplement: Supplementary file 1 — Supplementary Information [file 41467_2018_2874_MOESM1_ESM.pdf]

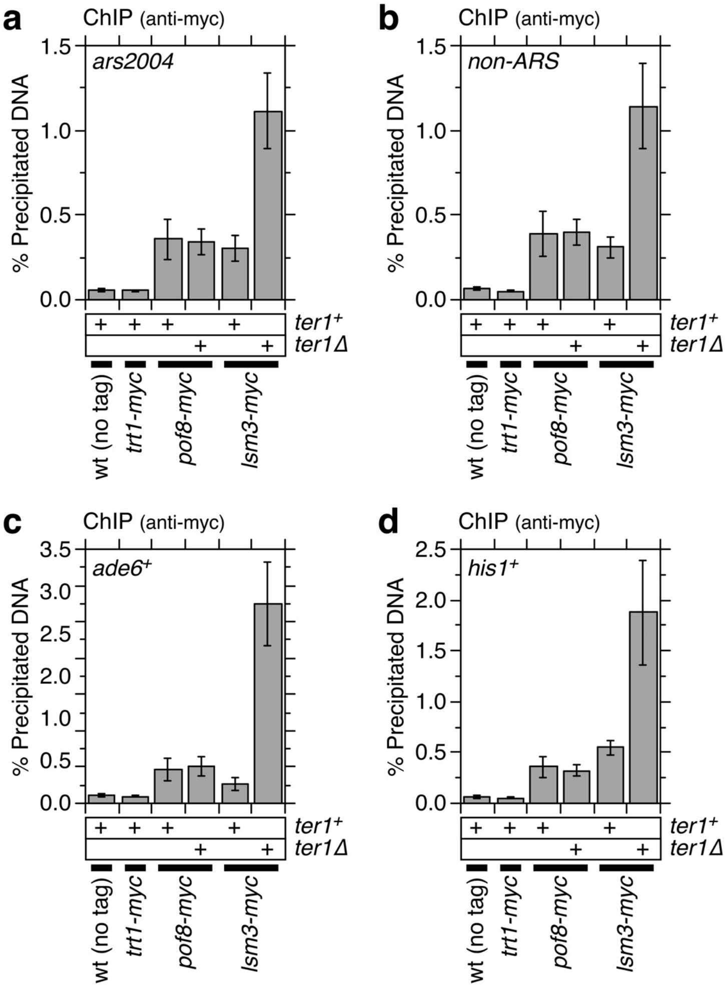

Supplementary Figure 1 Pof8 and Lsm3 bind to non-telomeric sites.
(**a-d**) Quantitative real-time PCR ChIP analysis to monitor association of Trt1, Pof8 and Lsm3 in indicated genetic backgrounds at (a) *ars2004*, (b) *non-ARS*, (c) $ade6^+$ and (d) $his1^+$. (See **Supplementary Table 4** for primers used in PCR.) Plots show mean values plus/minus SEM from at least 5 independent experiments. Raw data and statistical analysis are available in **Supplementary Dataset**.

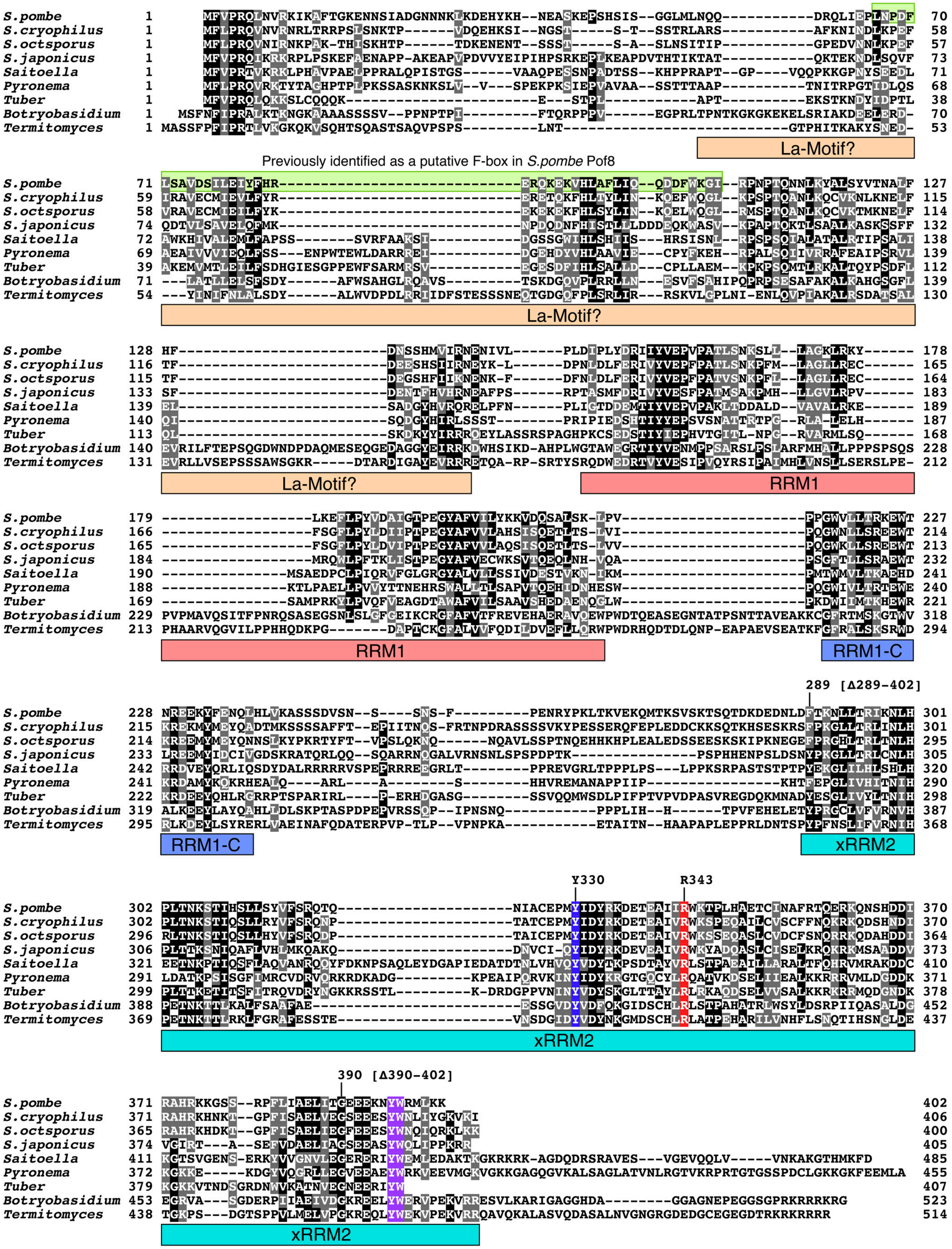
Previously identified as a putative F-box in S.pombe Pof8
S.pombe
S.cryophilus
S.octsporus
S.japonicus
Saitoella
Pyronema
Tuber
Botryobasidium
Termitomyces
La-Motif?
RRM1
RRM1-C
xRRM2
289 [Δ289-402]
Y330
R343
390 [Δ390-402]

Supplementary Figure 2 Sequence alignment of fungal Pof8-like proteins.

Proteins that show homology to fission yeast Pof8 were initially identified by PSH-BLAST (Position-Specific Iterated BLAST) at NCBI (https://blast.ncbi.nlm.nih.gov/Blast.cgi) after 3rd iterations using *S. pombe* Pof8 sequence. (Accession numbers for fungal Pof8-like proteins used in alignment are listed in **Supplementary Table 5**.) Multiple sequence alignment was generated by Clustal Omega[1]. Highly conserved residues within the xRRM domain that have been implicated in RNA recognition[2-4] are highlighted with colored background. Amino acid residues that show at least 50% conservation among aligned sequences are highlighted in black (identical residues) or gray (similar residues, grouped as GAVLI, FYW, CM, ST, KRH, DENQ, and P). Regions corresponding to putative La-motif, RRM1 and xRRM2 are marked. In addition, the region previously suggested to correspond to F-box in *S. pombe* Pof8[5] is indicated.

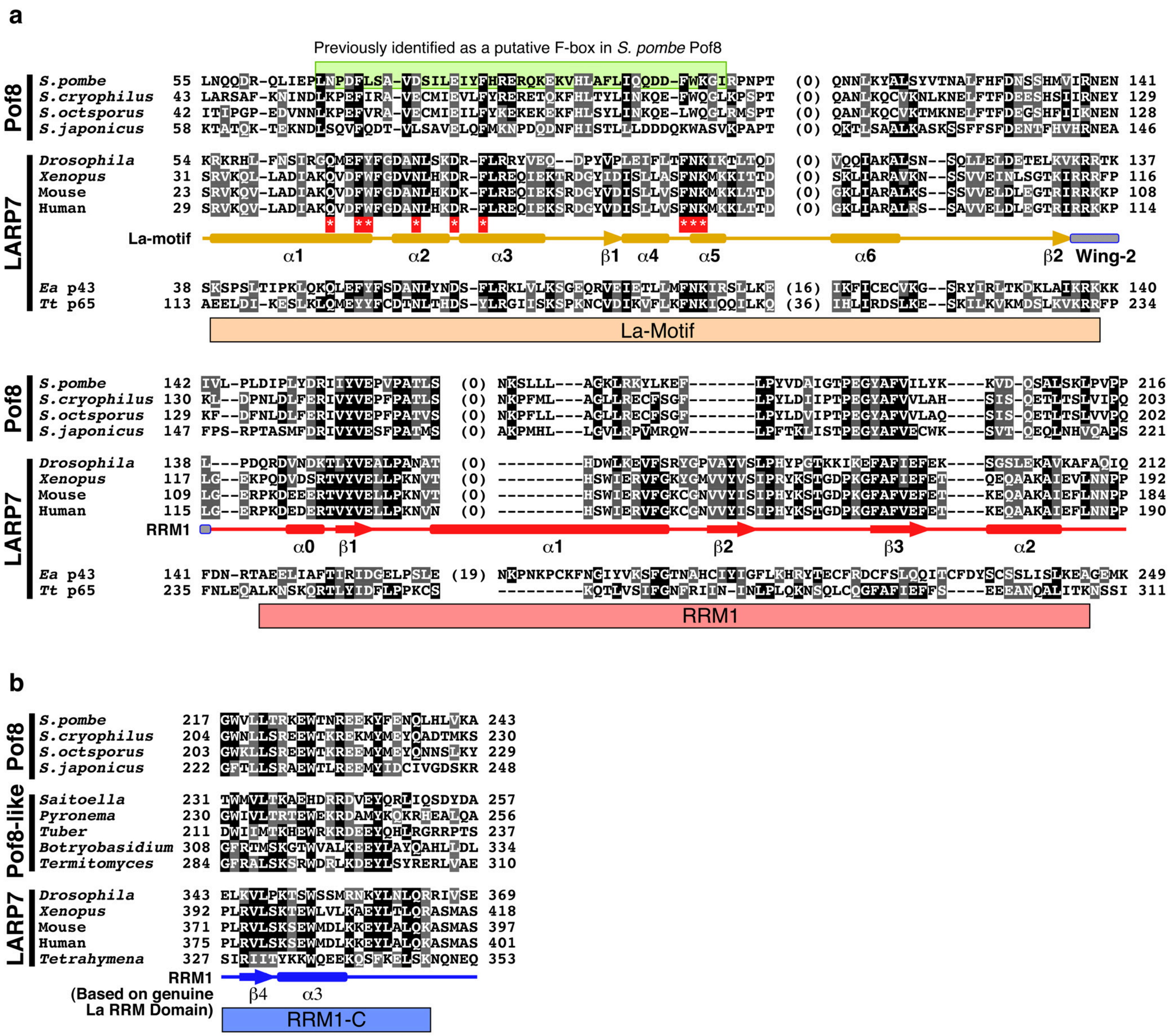

Supplementary Figure 3 Sequence alignment for the N-terminal region of Pof8 and LARP7 family proteins. (**a**) Sequence alignment of N-terminal regions that correspond to La-motif and RRM1 motif from Pof8 proteins from *Schizosacchromyces* species (*S. pombe*, *S. cryophilus*, *S. octsporus*, and *S. japonicus*), LARP7 proteins (*Drosophila*, *Xenopus*, mouse, and human), *Euplotes* p43, and *Tetrahymena* p65. Residues within the La-motif that are important for recognition of poly(U) sequence in human LARP7[6] are indicated with red colored square with Asterisk (*) mark. Amino acid residues that show at least 50% conservation among aligned sequences are highlighted in black (identical residues) or gray. The secondary structures for the human LARP7[6] La-motif and RRM1, and the putative F-box in *S. pombe* Pof8[5] are also indicated. (**b**) Alignment for additional region of homology C-terminal to RMM1 (RRM-C) among fission yeast Pof8, fungal Pof8-like proteins, and LARP7 family proteins from indicated species. This region of mammalian LARP7 has previously been identified to show homology to the α3 helix region of RRM1 in genuine La proteins[6]. The secondary structure of a genuine La protein for this region is also indicated.

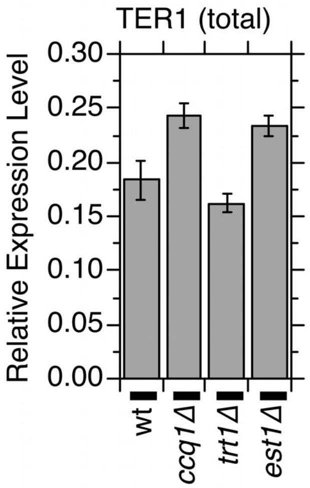

Supplementary Figure 4 TER1 RNA expression in *ccq1Δ*, *trt1Δ*, and *est1Δ* cells. TER1 RNA expression levels were normalized to *his1*$^{+}$ mRNA expression. Error bars correspond to SEM from at least 3 independent experiments, and raw data and statistical analysis are available in **Supplementary Dataset**.

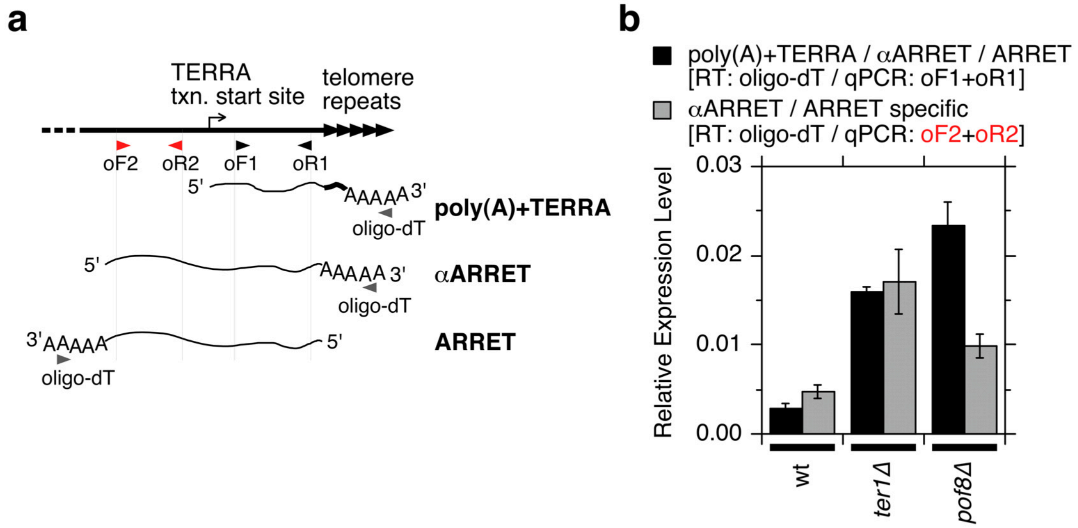

Supplementary Figure 5 Effect of *pof8Δ* on ARRET/αARRET expression.
(**a**) A schematic diagram indicating various poly(A)-tailed lncRNA species expressed at fission yeast telomere/sub-telomere regions[7,8]. Locations of primers used in RT reaction (oligo-dT) and subsequent quantitative PCR analysis (oF2 and oR2) to specifically monitor ARRET and αARRET, but not poly(A)+TERRA are indicated, along with primers that potentially detect all three type of poly(A)-tailed lncRNAs (oF1 and oR1) (**Supplementary Table 4**). (**b**) Expression levels of ARRET/αARRET is not greatly affected by *pof8Δ* mutation. Expression levels of telomeic transcripts were normalized to *his1*$^{+}$ mRNA. Error bars correspond to SEM from at least 5 independent experiments. Raw data and statistical analysis are available in **Supplementary Dataset**.

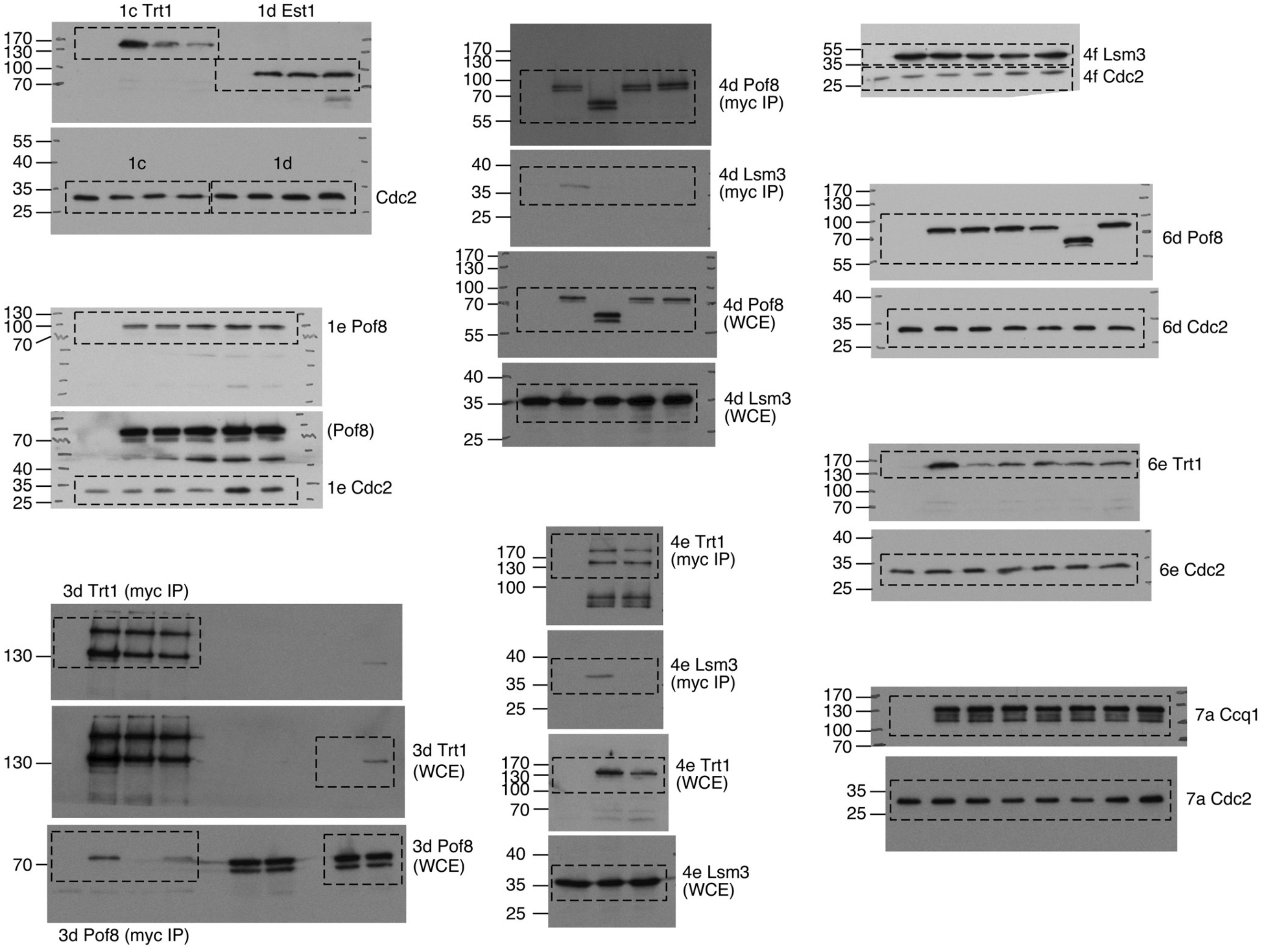

Supplementary Figure 6: Uncropped western blot gels for indicated main figures. Areas of gels shown in main figures are marked with dashed boxes. Sizes of protein molecular weight markers (kDa) are also indicated.

Supplementary Table 1: Fission yeast strains used in this study.

Figure	Strain		Full Genotype
1a	TN2411	wt	h- leu1-32 ura4-D18 his3-D1
	TN12119	pof8Δ	h+ leu1-32 ura4-D18 his3-D1 pof8Δ::kanMX6
	AM17672	pof8Δ trt1Δ	h- leu1-32 ura4-D18 his3-D1 trt1-2::his3$^+$ pof8Δ::kanMX6
	TN17261	pof8Δ rad52Δ*	h- leu1-32 ura4-D18 his3-D1 rad52Δ-D2::LEU2 pof8Δ::kanMX6
1b	TN2411	wt	h- leu1-32 ura4-D18 his3-D1
	TN12119	pof8Δ	h+ leu1-32 ura4-D18 his3-D1 pof8Δ::kanMX6
	AG14003	taz1Δ	h- leu1-32 ura4-D18 ade6-M210 his3-D1 taz1-2::ura4$^+$
	AM17697	pof8Δ taz1Δ	h- leu1-32 ura4-D18 his3-D1 taz1-2::ura4$^+$ pof8Δ::kanMX6
	YTC9370	rap1Δ	h+ leu1-32 ura4-D18 his3-D1 rap1::ura4$^+$
	AM17700	pof8Δ rap1Δ	h- leu1-32 ura4-D18 his3-D1 rap1::ura4$^+$ pof8Δ::kanMX6
	YTC8555	poz1Δ	h+ leu1-32 ura4-D18 his3-D1 poz1::natMX6
	AM17701	pof8Δ poz1Δ	h- leu1-32 ura4-D18 his3-D1 poz1::natMX6 pof8Δ::kanMX6
	YTC8432	rif1Δ	h+ leu1-32 ura4-D18 his3-D1 rif1Δ::ura4$^+$
	AM17695	pof8Δ rif1Δ	h- leu1-32 ura4-D18 his3-D1 rif1Δ::ura4$^+$ pof8Δ::kanMX6
	TN12119	pof8Δ	h+ leu1-32 ura4-D18 his3-D1 pof8Δ::kanMX6
1c	TN2411	trt1$^+$ (no tag)	h- leu1-32 ura4-D18 his3-D1
	TN10695	trt1-myc	h- leu1-32 ura4-D18 his3-D1 trt1$^+$::G8-13myc-natMX6
	TN10696	trt1-myc	h- leu1-32 ura4-D18 his3-D1 trt1$^+$::G8-13myc-natMX6
	TN16187	trt1-myc pof8Δ	h- leu1-32 ura4-D18 his3-D1 trt1$^+$::G8-13myc-natMX6 pof8Δ::kanMX6
	TN16188	trt1-myc pof8Δ	h- leu1-32 ura4-D18 his3-D1 trt1$^+$::G8-13myc-natMX6 pof8Δ::kanMX6
	TN16189	trt1-myc pof8Δ	h- leu1-32 ura4-D18 his3-D1 trt1$^+$::G8-13myc-natMX6 pof8Δ::kanMX6
	AM17632	trt1-myc ter1Δ	h+ leu1-32 ura4-D18 his3-D1 trt1$^+$::G8-13myc-kanMX6 ter1Δ[25-1135]::ura4$^+$-tk
	AM17633	trt1-myc ter1Δ	h+ leu1-32 ura4-D18 his3-D1 trt1$^+$::G8-13myc-kanMX6 ter1Δ[25-1135]::ura4$^+$-tk
1d	TN2411	est1$^+$ (no tag)	h- leu1-32 ura4-D18 his3-D1
	TN17157	est1-myc	h- leu1-32 ura4-D18 his3-D1 est1$^+$::G8-13myc-kanMX6
	TN17158	est1-myc	h- leu1-32 ura4-D18 his3-D1 est1$^+$::G8-13myc-kanMX6
	TN17171	est1-myc pof8Δ	h- leu1-32 ura4-D18 his3-D1 est1$^+$::G8-13myc-kanMX6 pof8Δ::kanMX6
	TN17172	est1-myc pof8Δ	h+ leu1-32 ura4-D18 his3-D1 est1$^+$::G8-13myc-kanMX6 pof8Δ::kanMX6
	TN17380	est1-myc ter1Δ	h+ leu1-32 ura4-D18 his3-D1 est1$^+$::G8-13myc-kanMX6 ter1-Δ[25-1135]::ura4$^+$-tk
	TN17381	est1-myc ter1Δ	h+ leu1-32 ura4-D18 his3-D1 est1$^+$::G8-13myc-kanMX6 ter1-Δ[25-1135]::ura4$^+$-tk
1e	TN2411	pof8$^+$ (no tag)	h- leu1-32 ura4-D18 his3-D1
	AM16931	pof8-myc	h+ leu1-32 ura4-D18 his3-D1 pof8$^+$::13myc-kanMX6
	AM16932	pof8-myc	h- leu1-32 ura4-D18 his3-D1 pof8$^+$::13myc-kanMX6
	AM17629	pof8-myc ter1Δ	h+ leu1-32 ura4-D18 his3-D1 ter1-Δ[25-1135]::ura4$^+$-tk pof8$^+$::13myc-kanMX6
	AM17630	pof8-myc ter1Δ	h+ leu1-32 ura4-D18 his3-D1 ter1-Δ[25-1135]::ura4$^+$-tk pof8$^+$::13myc-kanMX6
	AM17036	pof8-myc ccq1Δ	h- leu1-32 ura4-D18 his3-D1 ccq1Δ::hphMX pof8$^+$::13myc-kanMX6
	AM17037	pof8-myc ccq1Δ	h+ leu1-32 ura4-D18 his3-D1 ccq1Δ::hphMX pof8$^+$::13myc-kanMX6
	AM17026	pof8-myc trt1Δ	h- leu1-32 ura4-D18 his3-D1 trt1Δ::his3$^+$ pof8$^+$::13myc-kanMX6
	AM17027	pof8-myc trt1Δ	h- leu1-32 ura4-D18 ade6-M210 his3-D1 trt1Δ::his3$^+$ pof8$^+$::13myc-kanMX6
	AM17029	pof8-myc est1Δ	h+ leu1-32 ura4-D18 ade6-M210 his3-D1 est1Δ::kanMX6 pof8$^+$::13myc-kanMX6
	AM17030	pof8-myc est1Δ	h+ leu1-32 ura4-D18 ade6-M210 his3-D1 est1Δ::kanMX6 pof8$^+$::13myc-kanMX6
3a	TN2411	pof8$^+$ (no tag)	h- leu1-32 ura4-D18 his3-D1
	TN12132	pof8-myc	h- leu1-32 ura4-D18 ade6-M210 his3-D1 pof8$^+$::13myc-kanMX6
	AM16931	pof8-myc	h+ leu1-32 ura4-D18 his3-D1 pof8$^+$::13myc-kanMX6
	AM16932	pof8-myc	h- leu1-32 ura4-D18 his3-D1 pof8$^+$::13myc-kanMX6
	AM17036	pof8-myc ccq1Δ	h- leu1-32 ura4-D18 his3-D1 ccq1Δ::hphMX pof8$^+$::13myc-kanMX6
	AM17037	pof8-myc ccq1Δ	h+ leu1-32 ura4-D18 his3-D1 ccq1Δ::hphMX pof8$^+$::13myc-kanMX6
	AM17026	pof8-myc trt1Δ	h- leu1-32 ura4-D18 his3-D1 trtΔ::his3$^+$ pof8$^+$::13myc-kanMX6
	AM17027	pof8-myc trt1Δ	h- leu1-32 ura4-D18 ade6-M210 his3-D1 trt1Δ::his3$^+$ pof8$^+$::13myc-kanMX6
	AM17029	pof8-myc est1Δ	h+ leu1-32 ura4-D18 ade6-M210 his3-D1 est1Δ::kanMX pof8$^+$::13myc-kanMX6
	AM17030	pof8-myc est1Δ	h+ leu1-32 ura4-D18 ade6-M210 his3-D1 est1Δ::kanMX pof8$^+$::13myc-kanMX6
3b	TN2411	trt1$^+$ (no tag)	h- leu1-32 ura4-D18 his3-D1
	TN7706	trt1-myc	h- leu1-32 ura4-D18 his3-D1 trt1$^+$::G8-13myc-kanMX6
	TN10695	trt1-myc	h- leu1-32 ura4-D18 his3-D1 trt1$^+$::G8-13myc-natMX6
	TN10696	trt1-myc	h- leu1-32 ura4-D18 his3-D1 trt1$^+$::G8-13myc-natMX6
	TN16187	trt1-myc pof8Δ	h- leu1-32 ura4-D18 his3-D1 trt1$^+$::G8-13myc-natMX6 pof8Δ::kanMX6
	TN16188	trt1-myc pof8Δ	h- leu1-32 ura4-D18 his3-D1 trt1$^+$::G8-13myc-natMX6 pof8Δ::kanMX6
	TN16189	trt1-myc pof8Δ	h- leu1-32 ura4-D18 his3-D1 trt1$^+$::G8-13myc-natMX6 pof8Δ::kanMX6
	TN17597	trt1-myc pof8-Δ[289-402]	h+ leu1-32 ura4-D18 his3-D1 trt1+::G8-13myc-kanMX6 pof8-[Δ289-402]::natMX6

	TN17598	*trt1-myc pof8-Δ[289-402]*	*h+ leu1-32 ura4-D18 his3-D1 trt1+::G8-13myc-kanMX6 pof8-[Δ289-402]::natMX6*
3c	TN2411	*est1*$^{+}$ (no tag)	*h- leu1-32 ura4-D18 his3-D1*
	TN17157	*est1-myc*	*h- leu1-32 ura4-D18 his3-D1 est1*$^{+}$*::G8-13myc-kanMX6*
	TN17158	*est1-myc*	*h- leu1-32 ura4-D18 his3-D1 est1*$^{+}$*::G8-13myc-kanMX6*
	TN17171	*est1-myc pof8Δ*	*h- leu1-32 ura4-D18 his3-D1 est1*$^{+}$*::G8-13myc-kanMX6 pof8Δ::kanMX6*
	TN17172	*est1-myc pof8Δ*	*h+ leu1-32 ura4-D18 his3-D1 est1*$^{+}$*::G8-13myc-kanMX6 pof8Δ::kanMX6*
3d	AM17926	*pof8-PK*	*h- leu1-32 ura4-D18 his3-D1 pof8*$^{+}$*::12PK-hphMX6*
	AM17931	*pof8-PK trt1-myc*	*h+ leu1-32 ura4-D18 his3-D1 trt1*$^{+}$*::G8-13myc-kanMX6 pof8*$^{+}$*::12PK-hphMX6*
4a-b	TN2411	wt	*h- leu1-32 ura4-D18 his3-D1*
	TN12118	*pof8Δ*	*h- leu1-32 ura4-D18 his3-D1 pof8Δ::kanMX6*
	TN12119	*pof8Δ*	*h+ leu1-32 ura4-D18 his3-D1 pof8Δ::kanMX6*
4c	TN2411	*lsm3*$^{+}$ (no tag)	*h- leu1-32 ura4-D18 his3-D1*
	AM17121	*lsm3-myc*	*h- leu1-32 ura4-D18 his3-D1 lsm3*$^{+}$*::13myc-kanMX6*
	TN17147	*lsm3-myc*	*h+ leu1-32 ura4-D18 his3-D1 lsm3*$^{+}$*::13myc-kanMX6*
	AM17644	*lsm3-myc pof8Δ*	*h+ leu1-32 ura4-D18 his3-D1 pof8Δ::kanMX lsm3*$^{+}$*::13myc-kanMX6*
	AM17645	*lsm3-myc pof8Δ*	*h- leu1-32 ura4-D18 his3-D1 pof8Δ::kanMX6 lsm3*$^{+}$*::13myc-kanMX6*
4d	AM17755	*lsm3-PK*	*h+ leu1-32 ura4-D18 his3-D1 lsm3*$^{+}$*::12PK-natMX6*
	AM17746	*lsm3-PK pof8-myc*	*h+ leu1-32 ura4-D18 his3-D1 pof8*$^{+}$*::13myc-kanMX6 lsm3*$^{+}$*::12PK-natMX6*
	AM17946	*lsm3-PK pof8-Δ[289-402]-myc*	*h- leu1-32 ura4-D18 his3-D1 pof8-[Δ289-402] ::13myc-kanMX6 lsm3*$^{+}$*::12PK-natMX6*
	AM17949	*lsm3-PK pof8-myc ter1Δ*	*h+ leu1-32 ura4-D18 his3-D1 ter1Δ[25-1135]::ura4*$^{+}$*-tk pof8*$^{+}$*::13myc-kanMX6 lsm3*$^{+}$*::12PK-natMX6*
	AM17950	*lsm3-PK pof8-myc ter1Δ*	*h+ leu1-32 ura4-D18 his3-D1 ter1Δ[25-1135]::ura4*$^{+}$*-tk pof8*$^{+}$*::13myc-kanMX6 lsm3*$^{+}$*::12PK-natMX6*
4e	AM17755	*lsm3-PK*	*h+ leu1-32 ura4-D18 his3-D1 lsm3*$^{+}$*::12PK-natMX6*
	AM17744	*lsm3-PK trt1-myc*	*h+ leu1-32 ura4-D18 his3-D1 trt1*$^{+}$*::G8-13myc-kanMX6 lsm3*$^{+}$*::12PK-natMX6*
	AM17943	*lsm3-PK trt1-myc pof8Δ*	*h+ leu1-32 ura4-D18 his3-D1 trt1*$^{+}$*::G8-13myc-kanMX6 pof8Δ::kanMX6 lsm3*$^{+}$*::12PK-natMX6*
4f	TN2411	*lsm3*$^{+}$ (no tag)	*h- leu1-32 ura4-D18 his3-D1*
	AM17121	*lsm3-myc*	*h- leu1-32 ura4-D18 his3-D1 lsm3*$^{+}$*::13myc-kanMX6*
	TN17147	*lsm3-myc*	*h+ leu1-32 ura4-D18 his3-D1 lsm3*$^{+}$*::13myc-kanMX6*
	AM17644	*lsm3-myc pof8Δ*	*h+ leu1-32 ura4-D18 his3-D1 pof8Δ::kanMX6 lsm3*$^{+}$*::13myc-kanMX6*
	AM17645	*lsm3-myc pof8Δ*	*h- leu1-32 ura4-D18 his3-D1 pof8Δ::kanMX6 lsm3*$^{+}$*::13myc-kanMX6*
	TN17409	*lsm3-myc ter1Δ*	*h+ leu1-32 ura4-D18 his3-D1 ter1Δ[25-1135]::ura4*$^{+}$*-tk lsm3*$^{+}$*::13myc-kanMX6*
	TN17410	*lsm3-myc ter1Δ*	*h+ leu1-32 ura4-D18 his3-D1 ter1Δ[25-1135]::ura4*$^{+}$*-tk lsm3*$^{+}$*::13myc-kanMX6*
	TN17415	*lsm3-myc trt1Δ*	*h- leu1-32 ura4-D18 his3-D1 trt1Δ::his3*$^{+}$ *lsm3*$^{+}$*::13myc-kanMX6*
	TN17416	*lsm3-myc trt1Δ*	*h- leu1-32 ura4-D18 his3-D1 trt1Δ::his3*$^{+}$ *lsm3*$^{+}$*::13myc-kanMX6*
	AM17641	*lsm3-myc est1Δ*	*h+ leu1-32 ura4-D18 his3-D1 est1Δ::hphMX6 lsm3*$^{+}$*::13myc-kanMX6*
5a	TN17837	wt / No pld +B1	*h- leu1-32 his3-D1*
	TN17836	wt / No pld -B1	*h- leu1-32 his3-D1*
	TN17854	*pof8Δ* / Empty pld +B1	*h- leu1-32 ura4-D18 his3-D1 pof8Δ::kanMX //pREP42*
	TN17856	*pof8Δ* / Empty pld -B1	*h- leu1-32 ura4-D18 his3-D1 pof8Δ::kanMX //pREP42*
	TN17858	*pof8Δ* / TER1 OE pld +B1	*h- leu1-32 ura4-D18 his3-D1 pof8Δ::kanMX //pREP42-TER1*
	TN17859	*pof8Δ* / TER1 OE pld -B1	*h- leu1-32 ura4-D18 his3-D1 pof8Δ::kanMX //pREP42-TER1*
5b	TN17836	wt / No pld -B1	*h- leu1-32 his3-D1*
	AM17109	*ter1Δ* / No pld -B1	*h- leu1-32 ura4-D18 his3-D1 ter1Δ25-1135:ura4+:tk*
	TN17848	*ter1Δ* / TER1 OE pld -B1	*h- leu1-32 ura4-D18 his3-D1 ter1Δ25-1135:ura4+:tk //pREP41-TER1*
	TN17856	*pof8Δ* / Empty pld -B1	*h- leu1-32 ura4-D18 his3-D1 pof8Δ::kanMX //pREP42*
	TN17860	*pof8Δ* / TER1 OE pld -B1	*h- leu1-32 ura4-D18 his3-D1 pof8Δ::kanMX //pREP42*
5c	TN17836	wt (no tag) / No pld -B1	*h- leu1-32 his3-D1*
	TN17892	*trt1-myc* / Empty pld -B1	*h- leu1-32 ura4-D18 his3-D1 trt1*$^{+}$*::G8-13myc-natMX6 //pREP2*
	TN17916	*trt1-myc pof8Δ* / Empty pld -B1	*h- leu1-32 ura4-D18 his3-D1 trt1*$^{+}$*::G8-13myc-natMX6 pof8Δ::kanMX6 //pREP2*
	TN17908	*trt1-myc pof8Δ* / TER1 OE pld -B1	*h- leu1-32 ura4-D18 his3-D1 trt1*$^{+}$*::G8-13myc-natMX6 pof8Δ::kanMX6 //pREP42-TER1*
5d	TN17837	wt (no tag) / No pld +B1	*h- leu1-32 his3-D1*
	TN17890	*trt1-myc* / Empty pld +B1	*h- leu1-32 ura4-D18 his3-D1 trt1*$^{+}$*::G8-13myc-natMX6 pof8Δ::kanMX6 //pREP2*
	TN17891	*trt1-myc* / Empty pld +B1	*h- leu1-32 ura4-D18 his3-D1 trt1*$^{+}$*::G8-13myc-natMX6 pof8Δ::kanMX6 //pREP2*
	TN17892	*trt1-myc* / Empty pld -B1	*h- leu1-32 ura4-D18 his3-D1 trt1*$^{+}$*::G8-13myc-natMX6 pof8Δ::kanMX6 //pREP2*
	TN17893	*trt1-myc* / Empty pld -B1	*h- leu1-32 ura4-D18 his3-D1 trt1*$^{+}$*::G8-13myc-natMX6 pof8Δ::kanMX6 //pREP2*

	TN17914	trt1-myc pof8Δ /Empty pld +B1	h- leu1-32 ura4-D18 his3-D1 trt1^{+}::G8-13myc-natMX6 pof8Δ::kanMX6 //pREP2
	TN17915	trt1-myc pof8Δ /Empty pld +B1	h- leu1-32 ura4-D18 his3-D1 trt1^{+}::G8-13myc-natMX6 pof8Δ::kanMX6 //pREP2
	TN17906	trt1-myc pof8Δ / TER1 OE pld +B1	h- leu1-32 ura4-D18 his3-D1 trt1^{+}::G8-13myc-natMX6 pof8Δ::kanMX6 //pREP42-TER1
	TN17910	trt1-myc pof8Δ / TER1 OE pld +B1	h- leu1-32 ura4-D18 his3-D1 trt1^{+}::G8-13myc-natMX6 pof8Δ::kanMX6 //pREP42-TER1
	TN17908	trt1-myc pof8Δ / TER1 OE pld -B1	h- leu1-32 ura4-D18 his3-D1 trt1^{+}::G8-13myc-natMX6 pof8Δ::kanMX6 //pREP42-TER1
	TN17912	trt1-myc pof8Δ / TER1 OE pld -B1	h- leu1-32 ura4-D18 his3-D1 trt1^{+}::G8-13myc-natMX6 pof8Δ::kanMX6 //pREP42-TER1
6a	TN17580	pof8^{+}	h- leu1-32 ura4-D18 his3-D1 pof8^{+}::natMX6
	TN12119	pof8Δ	h+ leu1-32 ura4-D18 his3-D1 pof8Δ::kanMX6
	TN17582	pof8-Y330A	h- leu1-32 ura4-D18 his3-D1 pof8-Y330A::natMX6
	TN17584	pof8-R343A	h- leu1-32 ura4-D18 his3-D1 pof8-R343A::natMX6
	TN17587	pof8-Δ[390-402]	h- leu1-32 ura4-D18 his3-D1 pof8-[Δ390-402]::natMX6
	TN17586	pof8-Δ[289-402]	h- leu1-32 ura4-D18 his3-D1 pof8-[Δ289-402]::natMX6
6b	TN2411	pof8+ (no tag)	h- leu1-32 ura4-D18 his3-D1
	TN12132	pof8-myc	h- leu1-32 ura4-D18 ade6-M210 his3-D1 pof8^{+}::13myc-kanMX6
	AM16931	pof8-myc	h+ leu1-32 ura4-D18 his3-D1 pof8^{+}::13myc-kanMX6
	AM16932	pof8-myc	h- leu1-32 ura4-D18 his3-D1 pof8^{+}::13myc-kanMX6
	AM17098	pof8-Y330A-myc	h- leu1-32 ura4-D18 his3-D1 pof8-Y330A::13myc-kanMX6
	AM17616	pof8-R343A-myc	h- leu1-32 ura4-D18 his3-D1 pof8-R343A::13myc-kanMX6
	AM17123	pof8-Δ[390-402]-myc	h- leu1-32 ura4-D18 his3-D1 pof8-[Δ390-402]::13myc-kanMX6
	AM17618	pof8-Δ[289-402]-myc	h- leu1-32 ura4-D18 his3-D1 pof8-[Δ289-402] ::13myc-kanMX6
6c	AM16931	pof8-myc	h+ leu1-32 ura4-D18 his3-D1 pof8^{+}::13myc-kanMX6
	AM17098	pof8-Y330A-myc	h- leu1-32 ura4-D18 his3-D1 pof8-Y330A::13myc-kanMX6
	AM17616	pof8-R343A-myc	h- leu1-32 ura4-D18 his3-D1 pof8-R343A::13myc-kanMX6
	AM17123	pof8-Δ[390-402]-myc	h- leu1-32 ura4-D18 his3-D1 pof8-[Δ390-402]::13myc-kanMX6
	AM17618	pof8-Δ[289-402]-myc	h- leu1-32 ura4-D18 his3-D1 pof8-[Δ289-402] ::13myc-kanMX6
	TN12118	pof8Δ	h- leu1-32 ura4-D18 his3-D1 pof8Δ::kanMX6
	TN12119	pof8Δ	h+ leu1-32 ura4-D18 his3-D1 pof8Δ::kanMX6
6d	TN2411	pof8^{+} (no tag)	h- leu1-32 ura4-D18 his3-D1
	AM16931	pof8-myc	h+ leu1-32 ura4-D18 his3-D1 pof8^{+}::13myc-kanMX6
	AM16932	pof8-myc	h- leu1-32 ura4-D18 his3-D1 pof8^{+}::13myc-kanMX6
	AM17098	pof8-Y330A-myc	h- leu1-32 ura4-D18 his3-D1 pof8-Y330A::13myc-kanMX6
	AM17616	pof8-R343A-myc	h- leu1-32 ura4-D18 his3-D1 pof8-R343A::13myc-kanMX6
	AM17123	pof8-Δ[390-402]-myc	h- leu1-32 ura4-D18 his3-D1 pof8-[Δ390-402]::13myc-kanMX6
	AM17618	pof8-Δ[289-402]-myc	h- leu1-32 ura4-D18 his3-D1 pof8-[Δ289-402] ::13myc-kanMX6
	AM17629	pof8-myc ter1Δ	h+ leu1-32 ura4-D18 his3-D1 ter1Δ[25-1135]::ura4^{+}-tk pof8^{+}::13myc-kanMX6
	AM17630	pof8-myc ter1Δ	h+ leu1-32 ura4-D18 his3-D1 ter1Δ[25-1135]::ura4^{+}-tk pof8^{+}::13myc-kanMX6
6e	TN2411	trt1^{+} (no tag)	h- leu1-32 ura4-D18 his3-D1
	TN10695	trt1-myc	h- leu1-32 ura4-D18 his3-D1 trt1^{+}::G8-13myc-natMX6
	TN10696	trt1-myc	h- leu1-32 ura4-D18 his3-D1 trt1^{+}::G8-13myc-natMX6
	TN16187	trt1-myc pof8Δ	h- leu1-32 ura4-D18 his3-D1 trt1^{+}::G8-13myc-natMX6 pof8Δ::kanMX6
	TN16188	trt1-myc pof8Δ	h- leu1-32 ura4-D18 his3-D1 trt1^{+}::G8-13myc-natMX6 pof8Δ::kanMX6
	TN16189	trt1-myc pof8Δ	h- leu1-32 ura4-D18 his3-D1 trt1^{+}::G8-13myc-natMX6 pof8Δ::kanMX6
	TN17589	trt1-myc pof8-Y330A	h+ leu1-32 ura4-D18 his3-D1 trt1+::G8-13myc-kanMX6 pof8-Y330A::natMX6
	TN17590	trt1-myc pof8-Y330A	h+ leu1-32 ura4-D18 his3-D1 trt1+::G8-13myc-kanMX6 pof8-Y330A::natMX6
	TN17593	trt1-myc pof8-R343A	h+ leu1-32 ura4-D18 his3-D1 trt1+::G8-13myc-kanMX6 pof8-R343A::natMX6
	TN17594	trt1-myc pof8-R343A	h+ leu1-32 ura4-D18 his3-D1 trt1+::G8-13myc-kanMX6 pof8-R343A::natMX6
	TN17601	trt1-myc pof8-[Δ390-402]	h+ leu1-32 ura4-D18 his3-D1 trt1+::G8-13myc-kanMX6 pof8-[Δ390-402]::natMX6
	TN17602	trt1-myc pof8-[Δ390-402]	h+ leu1-32 ura4-D18 his3-D1 trt1+::G8-13myc-kanMX6 pof8-[Δ390-402]::natMX6
	TN17597	trt1-myc pof8-[Δ289-402]	h+ leu1-32 ura4-D18 his3-D1 trt1+::G8-13myc-kanMX6 pof8-[Δ289-402]::natMX6
	TN17598	trt1-myc pof8-[Δ289-402]	h+ leu1-32 ura4-D18 his3-D1 trt1+::G8-13myc-kanMX6 pof8-[Δ289-402]::natMX6
7a	TN2411	ccq1^{+} (no tag)	h- leu1-32 ura4-D18 his3-D1
	OR12046	ccq1-myc	h+ leu1-32 ura4-D18 his3-D1 ccq1^{+}::13myc-hphMX6
	OR12047	ccq1-myc	h+ leu1-32 ura4-D18 his3-D1 ccq1^{+}::13myc-hphMX6
	AM16956	ccq1-myc pof8Δ	h- leu1-32 ura4-D18 his3-D1 ccq1^{+}::13myc-hphMX6 pof8Δ::kanMX6
	AM16957	ccq1-myc pof8Δ	h+ leu1-32 ura4-D18 his3-D1 ccq1^{+}::13myc-hphMX6 pof8Δ::kanMX6
	AM17683	ccq1-myc ter1Δ	h+ leu1-32 ura4-D18 his3-D1 ccq1^{+}::13myc-kanMX6 ter1Δ[25-1135]::ura4^{+}:tk^{+}
	AM17685	ccq1-myc ter1Δ	h+ leu1-32 ura4-D18 his3-D1 ccq1^{+}::13myc-kanMX6 ter1Δ[25-1135]::ura4^{+}:tk^{+}
	TN17767	ccq1-myc pof8-Y330A	h- leu1-32 ura4-D18 his3-D1 ccq1^{+}::13myc-hphMX6 pof8-Y330A::natMX6
	TN17768	ccq1-myc pof8-Y330A	h- leu1-32 ura4-D18 his3-D1 ccq1^{+}::13myc-hphMX6 pof8-Y330A::natMX6
	TN17771	ccq1-myc pof8-R343A	h- leu1-32 ura4-D18 his3-D1 ccq1^{+}::13myc-hphMX6 pof8-R343A::natMX6

	TN17772	*ccq1-myc pof8-R343A*	*h- leu1-32 ura4-D18 his3-D1 ccq1*$^{+}$*::13myc-hphMX6 pof8-R343A::natMX6*
	TN17775	*ccq1-myc pof8-[Δ390-402]*	*h- leu1-32 ura4-D18 his3-D1 ccq1*$^{+}$*::13myc-hphMX6 pof8-[Δ390-402]::natMX6*
	TN17776	*ccq1-myc pof8-[Δ390-402]*	*h- leu1-32 ura4-D18 his3-D1 ccq1*$^{+}$*::13myc-hphMX6 pof8-[Δ390-402]::natMX6*
	TN17779	*ccq1-myc pof8-[Δ289-402]*	*h- leu1-32 ura4-D18 his3-D1 ccq1*$^{+}$*::13myc-hphMX6 pof8-[Δ289-402]::natMX6*
	TN17780	*ccq1-myc pof8-[Δ289-402]*	*h- leu1-32 ura4-D18 his3-D1 ccq1*$^{+}$*::13myc-hphMX6 pof8-[Δ289-402]::natMX6*
7b	TN2411	*ura4*$^{-}$	*h- leu1-32 ura4-D18 his3-D1*
	LK7299	*ura4*$^{+}$	*h- leu1-32 his3-D1*
	CF53	*telo::ura4*$^{+}$	*h*^{90} *leu1-32 ura4-D18 ade6-M210 his3-D1 otr1R(sphI):ade6*$^{+}$ *(cen1) his3*$^{+}$*:tel(1L) ura4*$^{+}$*:tel(2L)*
	CF17571	*pof8Δ telo::ura4*$^{+}$	*h*^{90} *leu1-32 ura4-D18 ade6-M210 his3-D1 pof8Δ::kanMX6 otr1R(sphI):ade6*$^{+}$ *(cen1) his3*$^{+}$*:tel(1L) ura4*$^{+}$*:tel(2L)*
	TN17822	*pof8-[Δ289-402] telo::ura4*$^{+}$	*h*^{90} *leu1-32 ura4-D18 ade6-M210 his3-D1 pof8-[Δ289-402]::13myc-kanMX6 otr1R(sphI):ade6+ (cen1) his3*$^{+}$*:tel(1L) ura4*$^{+}$*:tel(2L)*
	TN17823	*pof8-R343A telo::ura4*$^{+}$	*h*^{90} *leu1-32 ura4-D18 ade6-M210 his3-D1 pof8-R343A::13myc-kanMX6 otr1R(sphI):ade6*$^{+}$ *(cen1) his3*$^{+}$*:tel(1L) ura4*$^{+}$*:tel(2L)*
	TN17816	*ccq1Δ telo::ura4*$^{+}$	*h- leu1-32 ura4-D18 his3-D1 ccq1Δ::hphMX his3*$^{+}$*:tel(1L) ura4*$^{+}$*:tel(2L)*
7d	TN2409	wt	*h+ leu1-32 ura4-D18 his3-D1*
	TN2411	wt	*h- leu1-32 ura4-D18 his3-D1*
	AM17109	*ter1Δ*	*h- leu1-32 ura4-D18 his3-D1 ter1Δ[25-1135]::ura4*$^{+}$*-tk*
	AM17307	*ter1Δ*	*h+ leu1-32 ura4-D18 his3-D1 ter1Δ[25-1135]::ura4*$^{+}$*-tk*
	TN5345	*rap1Δ*	*h+ leu1-32 ura4-D18 his3-D1 rap1Δ::ura4*$^{+}$
	TN5346	*rap1Δ*	*h+ leu1-32 ura4-D18 his3-D1 rap1Δ::ura4*$^{+}$
	LK8667	*ccq1Δ*	*h- leu1-32 ura4-D18 his3-D1 ccq1Δ::hphMX*
	LK8668	*ccq1Δ*	*h- leu1-32 ura4-D18 his3-D1 ccq1Δ::hphMX*
	TN12118	*pof8Δ*	*h- leu1-32 ura4-D18 his3-D1 pof8Δ::kanMX6*
	TN12119	*pof8Δ*	*h+ leu1-32 ura4-D18 his3-D1 pof8Δ::kanMX6*
	AM16931	*pof8-myc*	*h+ leu1-32 ura4-D18 his3-D1 pof8*$^{+}$*::13myc-kanMX6*
	AM17098	*pof8-Y330A-myc*	*h- leu1-32 ura4-D18 his3-D1 pof8-Y330A::13myc-kanMX6*
	AM17616	*pof8-R343A-myc*	*h- leu1-32 ura4-D18 his3-D1 pof8-R343A::13myc-kanMX6*
	AM17123	*pof8-Δ[390-402]-myc*	*h- leu1-32 ura4-D18 his3-D1 pof8-[Δ390-402]::13myc-kanMX6*
	AM17618	*pof8-Δ[289-402]-myc*	*h- leu1-32 ura4-D18 his3-D1 pof8-[Δ289-402]::13myc-kanMX6*
S1	TN2411	*trt1*$^{+}$ (no tag)	*h- leu1-32 ura4-D18 his3-D1*
	TN10695	*trt1-myc*	*h- leu1-32 ura4-D18 his3-D1 trt1*$^{+}$*::G8-13myc-natMX6*
	AM16931	*pof8-myc*	*h+ leu1-32 ura4-D18 his3-D1 pof8*$^{+}$*::13myc-kanMX6*
	AM17630	*pof8-myc ter1Δ*	*h+ leu1-32 ura4-D18 his3-D1 ter1Δ[25-1135]::ura4*$^{+}$*-tk pof8*$^{+}$*::13myc-kanMX6*
	AM17121	*lsm3-myc*	*h- leu1-32 ura4-D18 his3-D1 lsm3*$^{+}$*::13myc-kanMX6*
	TN17409	*lsm3-myc ter1Δ*	*h+ leu1-32 ura4-D18 his3-D1 ter1Δ[25-1135]::ura4*$^{+}$*-tk lsm3*$^{+}$*::13myc-kanMX6*
S4	TN2411	*trt1*$^{+}$ (no tag)	*h- leu1-32 ura4-D18 his3-D1*
	LK8667	*ccq1Δ*	*h- leu1-32 ura4-D18 his3-D1 ccq1Δ::hphMX*
	LK8668	*ccq1Δ*	*h- leu1-32 ura4-D18 his3-D1 ccq1Δ::hphMX*
	TN10817	*trt1Δ*	*h- leu1-32 ura4-D18 ade6-M210 his3-D1 trt1Δ::his3*$^{+}$
	TN10818	*trt1Δ*	*h- leu1-32 ura4-D18 ade6-M210 his3-D1 trt1Δ::his3*$^{+}$
	TN10728	*est1Δ*	*h+ leu1-32 ura4-D18 ade6-M210 his3-D1 est1Δ::hphMX*
	TN10729	*est1Δ*	*h+ leu1-32 ura4-D18 ade6-M210 his3-D1 est1Δ::hphMX*
S5	TN2409	wt	*h+ leu1-32 ura4-D18 his3-D1*
	TN2411	wt	*h- leu1-32 ura4-D18 his3-D1*
	AM17109	*ter1Δ*	*h- leu1-32 ura4-D18 his3-D1 ter1Δ[25-1135]::ura4*$^{+}$*-tk*
	AM17307	*ter1Δ*	*h+ leu1-32 ura4-D18 his3-D1 ter1Δ[25-1135]::ura4*$^{+}$*-tk*
	TN12118	*pof8Δ*	*h- leu1-32 ura4-D18 his3-D1 pof8Δ::kanMX6*
	TN12119	*pof8Δ*	*h+ leu1-32 ura4-D18 his3-D1 pof8Δ::kanMX6*

*Rad52 was previously known as Rad22 in *S. pombe*, but it has been officially renamed to be more consistent with the name used in other organisms for orthologs of this protein.

Supplementary Table 2: Sources of various mutated and tagged alleles for fission yeast strains used in this study.

Previously published strains

Mutated & epitope-tagged alleles	Source
taz1Δ::ura4^{+}	[9]
rap1Δ::ura4^{+}	[10]
poz1Δ::natMX6	[11]
rif1Δ::ura4^{+}	[10]
trt1^{+}*::G8-13myc-kanMX6*	[12]
est1^{+}*::G8-13myc-kanMX6*	[12]
ccq1Δ::hphMX6	[13]
trt1Δ::his3^{+}	[14]
est1Δ::kanMX6	[15]
otr1R(sphI):ade6^{+} *(cen1) his3*$^{+}$*:tel(1L) ura4*$^{+}$*:tel(2L)*	[16]

New strains generated for this study

Mutated & epitope-tagged alleles	Comment
trt1^{+}*::G8-13myc-natMX6*	Marker swap of *trt1*$^{+}$*::G8-13myc:kanMX6* [12] by PCR-based method.
ccq1^{+}*::13myc-hphMX6*	Marker swap of *ccq1*$^{+}$*::13myc-kanMX6* [13] by PCR-based method.
ter1Δ[25-1135]::ura4^{+}*-tk*	Generated by two-step PCR-based method with plasmid 81 (Supplementary Table 3) and primers 1637, 1638, 1639, 1640 (Supplementary Table 4).
lsm3^{+}*::12PK-kanMX6*	Generated by two-step PCR-based method [17] with plasmid 973 (Supplementary Table 3) and primers 1686, 1689, 1687, 1690 (Supplementary Table 4).
lsm3^{+}*::13myc-kanMX6*	Generated by two-step PCR-based method [17] with plasmid 7 (Supplementary Table 3) and primers 1686, 1689, 1687, 1690 (Supplementary Table 4).
pof8Δ::kanMX6	Generated by two-step PCR-based method [17] with plasmid 7 (Supplementary Table 3) and primers 1383, 1390, 1386, and 1391 (Supplementary Table 4).
pof8^{+}*::12PK-kanMX6*	Generated by two-step PCR-based method [17] with plasmid 973 (Supplementary Table 3) and primers 1385, 1382, 1386, and 1391 (Supplementary Table 4).
pof8^{+}*::13myc-kanMX6*	Generated by two-step PCR-based method [17] with plasmid 7 (Supplementary Table 3) and primers 1385, 1382, 1386, and 1391 (Supplementary Table 4).
pof8-Y330A::13myc-kanMX6	Generated by two-step PCR-based method [18] with *pof8-myc* strain and primers 1699, 1655, 1656, 1386 (Supplementary Table 4).
pof8-R343A::13myc-kanMX6	Generated by two-step PCR-based method [18] with *pof8-myc* strain and primers 1699, 1658, 1386 (Supplementary Table 4).
pof8-[Δ390-402]::13myc-kanMX6	Generated by two-step PCR-based method [18] with *pof8-myc* strain and primers 1699, 1648, 1386 (Supplementary Table 4).
pof8-[Δ289-402]::13myc-kanMX6	Generated by two-step PCR-based method [18] with *pof8-myc* strain and primers 1699, 1647, 1386 (Supplementary Table 4).
pof8^{+}*::natMX6* (wt)	Removed epitope tag of *pof8-myc* strain via PCR-based method and primers 1699, 1724, 1285, 1386 (Supplementary Table 4).
pof8-Y330A::natMX6	Removed epitope tag of *pof8-Y330A-myc* strain via PCR-based method and primers 1699, 1724, 1285, 1386 (Supplementary Table 4).
pof8-R343A::natMX6	Removed epitope tag of *pof8-R343A-myc* strain via PCR-based method and primers 1699, 1724, 1285, 1386 (Supplementary Table 4).
pof8-[Δ390-402]::natMX6	Removed epitope tag of *pof8-[Δ390-402]-myc* strain via PCR-based method and primers 1699, 1726, 1285, 1386 (Supplementary Table 4).
pof8-[Δ289-402]::natMX6	Removed epitope tag of *pof8-[Δ289-402]-myc* strain via PCR-based method and primers 1699, 1725, 1285, 1386 (Supplementary Table 4).

Supplementary Table 3: Plasmids used in this study.

Plasmids used in fission yeast strain construction.

Stock # & plasmid name		Genes	Description
7	pFA6a-13myc-kanMX6 [17]	*kanMX6*; *ampR*	Used as a PCR template to generate 13xmyc-tagged strains and pof8Δ::kanMX6 strain.
973	pFA6a-12PK-kanMX6 [19]	*kanMX6; ampR*	Used as a PCR template to generate 12xPK-tagged strains.
81	pNR228 [20]	*ura4*$^{+}$*; adh1:tk*	Used as a PCR template to generate *ter1Δ[25-1135]::ura4*$^{+}$*-tk* strain with primers 1637, 1638, 1639, 1640 (Supplementary Table 4).
29	pREP2 [21]	*ura4*$^{+}$*; ampR*	Used as empty plasmid control.
30	pREP42 [21]	*ura4*$^{+}$*; ampR*	Used as empty plasmid control and for cloning *ter1*$^{+}$.
1245	pREP42-TER1	*ura4*$^{+}$*; ampR; ter1*$^{+}$	Primers 1826 and 1827 (Supplementary Table 4) were used to amplify *ter1*$^{+}$ from *S. pombe* genome and inserted into BamHI digested plasmid 30.
27	pREP41 [21]	*LEU2; ampR*	Used to clone *ter1*$^{+}$ gene.
1241	pREP41-TER1	*LEU2; ampR; ter1*$^{+}$	Primers 1826 and 1827 (Supplementary Table 4) were used to amplify *ter1*$^{+}$ from *S. pombe* genome and inserted into BamHI digested plasmid 27.

Additional fission yeast related plasmids.

Stock # & plasmid name		Genes	Description
254	pTELO [14]	Fission yeast telomere fragment; *ampR*	Carries a telomeric repeat fragment (ApaI-SacI) used in generating a telomere probe for Southern blot analysis.

Supplementary Table 4: DNA primers used in this study.

#	Primer Name	Primer Sequence (5' to 3')	Description
1383	Pof8-T2	GGAAAGACAAGGTCGTGGGTGCT	Anneals to *pof8* 5'UTR. Used to generate *pof8Δ::kanMX6* (sense).
1390	Pof8-KO(x)-B7	GGCAAGCTAAACAGATCTGGCGCTTTACTTCGCTCC TTAAAGTACGGTTTTC	Used to generate pof8ΔkanMX6 strain. Red letters anneal to kanMX6 on plasmid 7 (antisense) (Supplementary Table 3).
1386	Pof8-B5	GTGGTTCATGGTTTATTGGAATTTTTGCTG	Anneals to pof8 3' UTR. Used to generate *pof8Δ::kanMX6* (antisense).
1391	Pof8-KO/tag(y)-T8	GTCGATTCGATACTAACGCCGCCGTTTTCCTTTCTCT GGTAATACTAATTTG	Used to generate *pof8Δ::kanMX6* and 13Myc tagged *pof8* strains. Red letters anneal to pFA6a-13Myc-kanMX6 plasmid 7 (sense) (Supplementary Table 3).
1385	Pof8-T4	GCCCATGTATATAGACTATAGGAAGGACG	Anneals to *pof8*. Used to generate 13Myc tagged *pof8* strains (sense).
1382	Pof8-tag(x)-B1	GGGGATCCGTCGACCTGCAGCGTACGACTTTTTTAA CATACGCCAATAATTCTTTTCTTCTTCTC	Used to generate 13Myc tagged *pof8* strains. Red letters anneal to pFA6a-13Myc-kanMX6 plasmid 7 (antisense) (Supplementary Table 3).
1637	TER1ΔUTK-1F	CCACTCGGGACTTGCTTGTACTTTAACGG	Used to generate *ter1Δ[25-1135]::ura4*$^+$*-tk*$^+$ strain. Anneals upstream of *ter1* transcript (sense).
1638	TER1ΔUTK-1R	CGGGAGATGGGGGAGGCTAACTGACCTTCTAAGCA TGGGCGTTGCG	Used to generate *ter1Δ[25-1135]::ura4*$^+$*-tk*$^+$strain. Red letters anneal to plasmid 81 (antisense) (Supplementary Table 3).
1639	TER1ΔUTK-2F	GGCATATCAGCAAAGACTTTCTCAGCATTAACCTCTT GAGCGCGTTTTAGGTTTTTTTCAC	Used to generate *ter1Δ[25-1135]::ura4*$^+$*-tk*$^+$strain. Red letters anneal to plasmid 81 (sense) (Supplementary Table 3).
1640	TER1ΔUTK-2R	GATTCATCACTTTCTCAAAATTTTGAAACCGG	Used to generate *ter1Δ[25-1135]::ura4*$^+$*-tk*$^+$strain (antisense).
1602	Leu1Ter1-F	CCTGCAGCCCGGGGGATCCGTAAACGGAATATCCG CGATGAAA	Used to generate *leu1-32::[ter1+,leu1+]* strain. Red letters anneal to plasmid 37 (sense) (Supplementary Table 3).
1636	Leu1Ter1-R	GGCCGCTCTAGAACTAGTGGATCCACTTCATCTCTT CTAGTACGC	Used to generate *leu1-32::[ter1+,leu1+]* strain. Red letters anneal to plasmid 37 (antisense) (Supplementary Table 3).
1686	lsm3-T1	GATGATGAAGAGACCGATAAGGAC	Anneals to *lsm3*$^+$ (sense).
1689	lsm3-B1	GGATCCGTCGACCTGCAGCGTACGAATTACGAGGT GGAGCAATCAAAATAACC	Used to generate *lsm3*$^+$*::13Myc-kanMX6* strain. Red letters anneal to pFA6a-13Myc-kanMX6 plasmid 7(antisense) (Supplementary Table 3).
1687	lsm3-T2	CTGTCGATTCGATACTAACGCCGCCAGGAACGAATA AAATTACTATACAAAGC	Used to generate *lsm3*$^+$*::13Myc-kanMX6* strain. Red letters anneal to pFA6a-13Myc-kanMX6 plasmid 7 (sense) (Supplementary Table 3).
1690	lsm3-B2	CGTGAATGTTGGTTAACTTCG	Anneals to lsm3+ 3' UTR (antisense)
1655	pof8 Y330A-R	CGTCCTTCCTATAGTCTATAGCCATGGGCTCACATG CAATATTC	Used to generate Y330A mutation (red letters) in *pof8* (antisense).
1656	pof8 Y330A-F	GAATATTGCATGTGAGCCCATGGCTATAGACTATAG GAAGGACG	Used to generate Y330A mutation (red letters) in *pof8* (sense).
1657	pof8 R343A-R	GCATGAAGCGGTGTTTTCCAAGCTATTATCGCCTCT GTTTCGTCC	Used to generate R343A mutation (red letters) in *pof8* (antisense).
1648	pof8Δ390-402-R	CCGGGGATCCGTCGACCTGCAGCGTACGAGGTAAT TAGCTCAGCAATCAGGAATGGACGGC	Used to generate *pof8-Δ[390-402]-myc* strain. Red letters anneal to pFA6a-kanMX6 plasmid.
1647	pof8Δ289-402-R	CGGGGATCCGTCGACCTGCAGCGTACGAATCCAAA TTATCTTCATCTTTATCAGTTTG	Used to generate *pof8-Δ[289-402]-myc* strain. Red letters anneal to pFA6a-13Myc-kanMX6 plasmid 7 (Supplementary Table 3).
1285	Tpz1-T54	CGCGCCACTTCTAAATAAGCGAATTTCTT	Used to remove 13myc tag from *pof8-myc* strains.
1699	pof8-TC	CCTTTCCTGAAAATCGTTATCCC	Anneals to *pof8* (sense).
1724	pof8ΔMyc	GAAATTCGCTTATTTAGAAGTGGCGCGTTACTTTTTT AACATACGCCAATAATTCTTTTC	Used to remove 13myc tag from full length *pof8-myc* strains.
1725	pof8Δ289Myc	GAAATTCGCTTATTTAGAAGTGGCGCGTTAATCCAAA TTATCTTCATCTTTATCAGTTTG	Used to remove 13myc tag from *pof8-Δ[289-402]-myc* strain.
1726	pof8Δ390Myc	GAAATTCGCTTATTTAGAAGTGGCGCGTTAGGTAATT AGCTCAGCAATCAGGAATGGACG	Used to remove 13myc tag from *pof8-Δ[390-402]-myc* strain.

1826	pREPter1F	CATATGTCGACTCTAGAGGATCCTTATACTCAACG CAACGCCC	Used to clone TER1 gene in pREP41 or pREP42 (Supplementary Table 3). Red sequence contains homology to plasmids 27, 29, or 30.
1827	pREPter1R	CATTCCTTTTACCCGGGGATCCACTTCATCTCTTCT AGTACGC	Used to clone TER1 gene in pREP41 or pREP42 (Supplementary Table 3). Red sequence contains homology to plasmids 27, 29, or 30.
637	jk380 (TEL-#1)	TATTTCTTTATTCAACTTACCGCACTTC	Used in qPCR in telomere ChIP experiments [22].
638	jk381 (TEL-#2)	CAGTAGTGCAGTGTATTATGATAATTAAAATGG	Used in qPCR in telomere ChIP experiments [22].
935	ars2004-66-F	CGGATCCGTAATCCCAACAA	Used in qPCR in *ars2004* ChIP experiments [23].
936	ars2004-66-R	TTTGCTTACATTTTCGGGAACTTA	Used in qPCR in *ars2004* ChIP experiments [23].
941	non-ARS-70-F	TACGCGACGAACCTTGCATAT	Used in qPCR in *non-ARS* (~30 kb from *ars2004*) ChIP experiments [23].
942	non-ARS-70-R	TTATCAGACCATGGAGCCCATT	Used in qPCR in *non-ARS* (~30 kb from *ars2004*) ChIP experiments [23].
633	Ade6-3	TGATGGAGGACGTGAGCACATTGA	Used in qPCR in $ade6^+$ ChIP experiments.
634	Ade6-4	TTGAATGCATCGCAGAGTTGCAGG	Used in qPCR in $ade6^+$ ChIP experiments.
1779	his1.1	CGAAGACGTGCTTCAGCGA	Used in $his1^+$ ChIP experiments, and RT and qPCR for $his1^+$ expression studies [24].
1780	his1.2	TGTCCACCTCGGAATCACTG	Used in $his1^+$ ChIP experiments, and RT and qPCR for $his1^+$ expression studies [24].
1016	3'TER1-B1	GATCCATGGATCTCACGTAATG	Used in RT for TER1.
1015	5'TER1-T1	CAGTGTACGTGAGTCTTCTGCCTT	Used in qPCR for TER1.
1017	275-Ter1	CAAAAATTCGTTGTGATCTGACAAGC	Used in qPCR for TER1.
1678	BLoli1275	CGGAAACGGAATTCAGCATGT	Used in RT and qPCR for un-spliced TER1 [25].
1679	Bloli1020	CAAA CAATAATGAACGTCCTG	Used in qPCR for un-spliced TER1 [25].
1779	his1.1	CGAAGACGTGCTTCAGCGA	Used in RT and qPCR for $his1^+$ [24].
1780	his1.2	TGTCCACCTCGGAATCACTG	Used in qPCR for $his1^+$ [24].
1772	odT	TTTTTTTTTTTTTTTTTTTTT	Used in RT for polyA-TERRA/ARRET [8].
1773	oF1	GAAGTTCACTCAGTCATAATTAATTGGGTAACGGAG	Used in qPCR for polyA-TERRA/ARRET [8].
1775	oR1	GGGCCCAATAGTGGGGGCATTGTATTTGTG	Used in qPCR for polyA-TERRA/ARRET [8].
1774	oF2	GGTTGAATTGAGCGTGGTAGG	Used in qPCR for polyA-ARRET [8].
1776	oR2	ACTTACTGCACCCTAACGCA	Used in qPCR for polyA-ARRET [8].

Supplementary Table 5: Sources of sequences used in bioinformatic analysis.

**Protein Name**	**GenBank Acc. No.**	**Source**
Pof8	BAB60688	*Schizosaccharomyces pombe*
Pof8	EPX72631	*Schizosaccharomyces octosporus* yFS286
Pof8	EPY52790	*Schizosaccharomyces cryophilus* OY26
Pof8	EEB09384.1	*Schizosaccharomyces japonicus* yFS275
LARP7	NP_524795	*Drosophila melanogaster*
LARP7	NP_001039168	*Xenopus tropicalis*
LARP7	AFE61891	*Mus musculus*
LARP7	AAH66945	*Homo sapiens*
p43*	AF307939	*Euplotes aediculatus*
p65	AY280524	*Tetrahymena thermophila*
hypothetical protein G7K_2053-t1	GAO47857	*Saitoella complicata* NRRL Y-17804
similar to Pof8	CCX33746	*Pyronema omphalodes* CBS 100304
hypothetical protein	XP_002836370	*Tuber melanosporum* Mel28 (black truffle mushroom)
hypothetical protein BOTBODRAFT_187311	KDQ15495	*Botryobasidium botryosum* FD-172 SS1
Pof8	KNZ72704	*Termitomyces* sp. J132 (mushroom)

***NOTE:** Final protein sequence surrounding open reading frameshift site was adjusted as originally proposed by Aigner *et al.* [26].

SUPPLEMENTARY REFERENCES

1. Sievers, F. et al. Fast, scalable generation of high-quality protein multiple sequence alignments using Clustal Omega. *Mol Syst Biol* **7**, 539 (2011).
2. Singh, M. et al. Structural basis for telomerase RNA recognition and RNP assembly by the holoenzyme La family protein p65. *Mol Cell* **47**, 16-26 (2012).
3. Singh, M., Choi, C.P. & Feigon, J. xRRM: a new class of RRM found in the telomerase La family protein p65. *RNA Biol* **10**, 353-9 (2013).
4. Eichhorn, C.D., Chug, R. & Feigon, J. hLARP7 C-terminal domain contains an xRRM that binds the 3' hairpin of 7SK RNA. *Nucleic Acids Res* **44**, 9977-9989 (2016).
5. Schmidt, M.W., McQuary, P.R., Wee, S., Hofmann, K. & Wolf, D.A. F-box-directed CRL complex assembly and regulation by the CSN and CAND1. *Mol Cell* **35**, 586-97 (2009).
6. Uchikawa, E. et al. Structural insight into the mechanism of stabilization of the 7SK small nuclear RNA by LARP7. *Nucleic Acids Res* **43**, 3373-88 (2015).
7. Bah, A., Wischnewski, H., Shchepachev, V. & Azzalin, C.M. The telomeric transcriptome of *Schizosaccharomyces pombe*. *Nucleic Acids Res* **40**, 2995-3005 (2012).
8. Moravec, M. et al. TERRA promotes telomerase-mediated telomere elongation in *Schizosaccharomyces pombe*. *EMBO Rep* **17**, 999-1012 (2016).
9. Cooper, J.P., Nimmo, E.R., Allshire, R.C. & Cech, T.R. Regulation of telomere length and function by a Myb-domain protein in fission yeast. *Nature* **385**, 744-7 (1997).
10. Kanoh, J. & Ishikawa, F. spRap1 and spRif1, recruited to telomeres by Taz1, are essential for telomere function in fission yeast. *Curr Biol* **11**, 1624-30 (2001).
11. Khair, L., Subramanian, L., Moser, B.A. & Nakamura, T.M. Roles of heterochromatin and telomere proteins in regulation of fission yeast telomere recombination and telomerase recruitment. *J Biol Chem* **285**, 5327-37 (2010).
12. Webb, C.J. & Zakian, V.A. Identification and characterization of the *Schizosaccharomyces pombe* TER1 telomerase RNA. *Nat Struct Mol Biol* **15**, 34-42 (2008).
13. Moser, B.A., Subramanian, L., Khair, L., Chang, Y.T. & Nakamura, T.M. Fission yeast $Tel1^{ATM}$ and $Rad3^{ATR}$ promote telomere protection and telomerase recruitment. *PLoS Genet* **5**, e1000622 (2009).
14. Nakamura, T.M. et al. Telomerase catalytic subunit homologs from fission yeast and human. *Science* **277**, 955-9 (1997).
15. Beernink, H.T., Miller, K., Deshpande, A., Bucher, P. & Cooper, J.P. Telomere maintenance in fission yeast requires an Est1 ortholog. *Curr Biol* **13**, 575-80 (2003).
16. Nimmo, E.R., Pidoux, A.L., Perry, P.E. & Allshire, R.C. Defective meiosis in telomere-silencing mutants of *Schizosaccharomyces pombe*. *Nature* **392**, 825-8 (1998).
17. Bähler, J. et al. Heterologous modules for efficient and versatile PCR-based gene targeting in *Schizosaccharomyces pombe*. *Yeast* **14**, 943-51 (1998).
18. Krawchuk, M.D. & Wahls, W.P. High-efficiency gene targeting in *Schizosaccharomyces pombe* using a modular, PCR-based approach with long tracts of flanking homology. *Yeast* **15**, 1419-27 (1999).
19. Gadaleta, M.C., Iwasaki, O., Noguchi, C., Noma, K. & Noguchi, E. New vectors for epitope tagging and gene disruption in *Schizosaccharomyces pombe*. *Biotechniques* **55**, 257-63 (2013).
20. Sivakumar, S., Porter-Goff, M., Patel, P.K., Benoit, K. & Rhind, N. *In vivo* labeling of fission yeast DNA with thymidine and thymidine analogs. *Methods* **33**, 213-9 (2004).
21. Basi, G., Schmid, E. & Maundrell, K. TATA box mutations in the *Schizosaccharomyces pombe nmt1* promoter affect transcription efficiency but not the transcription start point or thiamine repressibility. *Gene* **123**, 131-6 (1993).
22. Kanoh, J., Sadaie, M., Urano, T. & Ishikawa, F. Telomere binding protein Taz1 establishes Swi6 heterochromatin independently of RNAi at telomeres. *Curr Biol* **15**, 1808-19 (2005).

23. Hayashi, M. et al. Genome-wide localization of pre-RC sites and identification of replication origins in fission yeast. *EMBO J* **26**, 1327-39 (2007).
24. Fujita, I., Tanaka, M. & Kanoh, J. Identification of the functional domains of the telomere protein Rap1 in *Schizosaccharomyces pombe*. *PLoS One* **7**, e49151 (2012).
25. Tang, W., Kannan, R., Blanchette, M. & Baumann, P. Telomerase RNA biogenesis involves sequential binding by Sm and Lsm complexes. *Nature* **484**, 260-4 (2012).
26. Aigner, S. et al. *Euplotes* telomerase contains an La motif protein produced by apparent translational frameshifting. *EMBO J* **19**, 6230-9 (2000).
