# Supplementary material for: LARP7-like protein Pof8 regulates telomerase assembly and poly(A)+TERRA expression in fission yeast
Source: Nat Commun. 2018 Feb 8;9:586. doi: 10.1038/s41467-018-02874-0 (PMC5805695; doi:10.1038/s41467-018-02874-0)
Supplement: Supplementary file 3 — Description of Additional Supplementary Files [file 41467_2018_2874_MOESM3_ESM.pdf]

Description of Additional Supplementary Files

File Name: Supplementary Data 1
Description: Raw data and statistical analysis.
